# The Benefits of Integrative Medicine in the Management of Chronic Pain: A Review

**DOI:** 10.7759/cureus.29963

**Published:** 2022-10-05

**Authors:** Hirak Trivedi, Tara A Avrit, Leah Chan, De Mauria Burchette, Rajni Rathore

**Affiliations:** 1 Medical Student, Saint James School of Medicine, Arnos Vale, VCT; 2 School of Medicine, Saint James School of Medicine, Anguilla, AIA; 3 Medical Education, Saint James School of Medicine, Arnos Vale, VCT

**Keywords:** opioid abuse, osteopathic manipulative medicine, acupuncture, yoga therapy, alternative medical therapies, chronic pain management

## Abstract

Chronic pain is a debilitating condition that affects many individuals throughout their daily lives. While it is common to treat chronic pain with pharmaceutical treatments, an approach that has also shown great benefits is the use of integrative medicine, such as massage therapy, osteopathic and spinal manipulation, acupuncture, and yoga. The keywords “integrative medicine,” “pain,” “chronic pain,” and “pain management” with the use of the Boolean operators “AND,” “OR,” and “NOT” were used to identify relevant studies discussing the effectiveness of alternative medicine in the treatment of chronic pain. Massage therapy uses different forms of pressing, rubbing, and moving of muscles and other soft tissues, and has shown short-term benefits for chronic pain relief. Osteopathic and spinal manipulation is mainly used in treating muscles, tendons, and bone pain due to worn-out joints, torn ligaments, and more. Acupuncture involves penetrating the skin with thin needles which are activated through gentle and specific movements. According to our review, acupuncture and massage therapy are effective for short-term treatment, lasting three to five months for chronic pain. Yoga involves various physical, mental, and spiritual practices or disciplines that have shown beneficial results in the treatment of chronic pain. Combining yoga with physical therapy has shown significant benefits. This review aims to describe the benefits and uses of integrative medicine in the treatment of chronic pain.

## Introduction and background

Chronic pain, described as pain lasting longer than three to six months, can be a debilitating condition with a significant socioeconomic impact. Although pharmacologic approaches are often used for alleviating chronic pain, recently, there has been a reluctance to prescribe opioids for chronic non-cancer pain because of concerns regarding tolerance, dependence, and addiction. Consequently, there has been an increased interest in integrative medicine strategies to help manage pain and reduce reliance on prescription opioids to manage pain [1].

In integrative medicine, a patient receives a non-traditional course of treatment that allows the healing process. This approach to medicine is patient-centered, healing-oriented, and uses therapeutic approaches that originate from both conventional and alternative medicine [2]. This type of medicine takes part in the importance of therapeutic relationships that address mind, body, and spiritual needs, while still coordinating care with a team of medical physicians [2].

Through personalized care, integrative medicine goes beyond the treatment of symptoms to address all the causes of an illness. In doing so, the patient’s immediate health, needs, and the effects of the long-term and complex interplay among biological, behavioral, psychosocial, and environmental influences are taken into account [3].

All forms of alternative therapy are effective methods of treatment for chronic pain. When compared to traditional treatments for nonspecific chronic pain such as non-steroidal anti-inflammatory drugs (NSAIDs), opioids, and steroids, integrative techniques present with far fewer adverse effects. For example, Bindu et al. reported that the success of NSAIDs comes at a significant cost due to adverse drug reactions related to organ damage, including, but not limited to, gastric ulcers, hepatotoxicity, intracerebral hemorrhage, and the risk of respiratory inflammation and infection [4]. Moreover, there is ample evidence demonstrating opioid-induced gastrointestinal adverse effects along with notable addictive potential, as well as the potentially detrimental immunosuppression associated with steroid use that can lead to pathologies such as fungal infections [5,6]. Due to the potential adverse toxicities related to traditional treatments for chronic pain, integrative medicine techniques such as massage, osteopathic spinal manipulation, acupuncture, and yoga are extremely relevant in the treatment of chronic pain and should be considered a leading treatment option.

This article offers a brief review of integrative medical therapies used to treat chronic pain, including massage therapy, osteopathic and spinal manipulation, acupuncture, and yoga. Integrative medicine is an evolving field and can be highly beneficial in the treatment of chronic pain. Because first-line treatments for chronic pain can cause several lifelong side effects, it is crucial to study the impacts of integrative medicine on chronic pain.

## Review

Methodology

In January 2021, a review was conducted using the resources available at the Saint James School of Medicine Library as well as additional resources such as PubMed and EBSCO electronic databases. The keywords “integrative medicine,” “pain,” “chronic pain,” and “pain management” with the use of the Boolean operators “AND,” “OR,” and “NOT” were used to identify relevant studies discussing the effectiveness of alternative medicine in the treatment of chronic pain.

The following inclusion criteria were applied: (1) A scholarly reviewed journal/source such as PubMed and the National Library of Medicine. (2) Recent articles published within the past seven years, at the beginning date of the research. (3) Articles published in the English language. (4) Articles that only included human participants.

The following exclusion criteria were applied: (1) Did not include participants under the age of 18. (2) Studies conducted in an inpatient setting. (3) Observational studies.

The outcome measures included the change in the degree of pain, quality of life before and after the intervention, and side effects. These outcomes were measured using various pain scales by different evaluators. These pain scales were typically measured on a range to assess pain levels. Studies that showed a decrease on pain scales were included. In total, 15 out of 27 articles were selected for the review based on the inclusion and exclusion criteria.

Massage therapy

Massage therapy is a practice that dates back thousands of years. There are many types of massage therapy, including pressing, rubbing, and moving muscles and other soft tissues of the body. The aim is to increase the flow of blood and oxygen to the massaged area. Massage therapy involves manipulating the muscles and other soft tissues of the body. In the United States, massage therapy is sometimes seen as a part of conventional medicine [7].

A randomized clinical trial was performed over a four-week treatment period consisting of participants aged 20 to 64 years to determine the optimal dose of massage therapy for neck pain [8]. The study reported no clinically significant differences in the phase one treatment group at 12 or 26 weeks. Outcomes were measured at baseline, 12 weeks, and 26 weeks using the self-reported Neck Disability Index which measures neck-related pain and dysfunction on a 1-10 scale [8]. The Neck Disability Index is a self-report questionnaire where patients assess the daily disabilities they face due to their neck pain [8]. Clinically meaningful improvements were defined as greater than or equal to a five-point decrease in dysfunction and greater than or equal to a 30% decrease in pain from baseline. In phase one, the study had a total of 228 participants randomized into one of six groups [8]: one wait-list (control group) and a four-week massage intervention dose of either two 40-minute massages, three 30-minute massages, one 60-minute massage, two 60-minute massages, or three 60-minute massages per week. The 179 intervention participants were divided into either phase two of one additional 60-minute booster massage for six weeks or no additional massages [8]. Those who received the phase two booster dose had improvements in both pain and dysfunction at 12 weeks but were non-significant at 26 weeks [8]. Further subgroup analysis found the booster dose was only effective among those initially randomized into one of the 60-minute massage groups. A limitation of this study was that the prolonged group (phase two booster dose) did not have control, and any improvements could have been associated with the natural history of neck pain. The study concluded that further investigations for neck pain should use multiple 60-minute treatments for the first four weeks and booster doses of at least 60-minute treatments for six weeks [8]. The study suggested that future studies include a comparison group of persons seeking conventional medical care to assess the longer-term effects of massage.

A pilot randomized clinical trial was performed on breast cancer surgery patients to determine the value of myofascial massage to address pain and mobility limitations [9]. Results showed favorable changes in the intervention group. The study included 21 women with pain and mobility limitations three to 18 months following breast surgery, ranging from simple mastectomy, partial mastectomy, mastectomy, and axillary node dissection [9]. The participants were randomized into two groups of two 30-minute-per-week massages over an eight-week period. One group received myofascial massage sessions specific to the breast, chest, and shoulder of the affected side. Various techniques were specifically developed for the study to reduce pain, inflammation, and tissue sensitivity, while also increasing mobility by breaking up scar tissue [9]. The control group received 16 relaxation massages such as Swedish relaxation massages that involved light kneading and stroking while avoiding the breast, chest, and shoulder area. Participants completed a questionnaire at the beginning and end of the study that asked about pain, mobility, and quality of life [9]. The main outcome measures included change in self-reported pain, self-reported mobility, and three quality-of-life questions. At baseline, intervention and control participants were similar in demographic and medical characteristics, pain and mobility ratings, and quality of life [9]. Results showed that intervention participants had more favorable changes in pain, mobility, and general health after eight weeks. All 21 participants reported that receiving a massage was an overall positive experience. The study concluded that myofascial massage is a promising treatment to address chronic pain and mobility limitations post-breast cancer surgery [9]. The study suggested that future studies increase participant groups and assess objective outcomes, as well as patient-centered outcomes, along with understanding the impact of massage therapy on long-term outcomes [9].

In 2015, a review examined 25 different studies concerning massage therapy and patients with chronic lower back pain [10]. The study reported that patients with acute, subacute, and chronic lower back pain who received massage therapy showed improvements in the short term, as evidenced by improved function and decreased pain. To determine the optimal dosing for massage therapy and chronic lower back pain randomized controlled trials were conducted to systematically evaluate a proper dose [10]. One of the randomized controlled trials conducted for a four-week period included patients with chronic neck pain who were randomized into a control group and a dosing level of one to five [10]. Dosing differed by a 30-minute massage session, two to three times a week, compared to one 60-minute session, two to three times a week. The 60-minute massage therapy, two to three times a week, showed improved function and decreased pain [10]. In the 25 different studies on massage therapy that were analyzed, there were no major adverse reactions to massage therapy, and patients mostly benefited from massage therapy in the short term compared to in the long term [10].

Osteopathic and spinal manipulation

Osteopathic manipulative treatment (OMT), also called osteopathic manipulative therapy or osteopathic manipulation, is a hands-on treatment method [11]. OMT is mainly used in the treatment of mechanical pain such as muscle, tendon, or bone pain due to structural imbalances like worn-out joints, torn or lax ligaments, and a wide range of health conditions [11]. OMT treatment encourages the body to heal itself by ensuring that the bones and muscles are aligned and balanced properly. OMT can be used to treat a wide variety of common ailments, including, but not limited to, headaches, arthritis, stress injuries, sports injuries, and pain in areas such as the lower back, neck, shoulders, and knees [11].

Lower back pain is one of the most prevalent musculoskeletal conditions in the United States, mainly affecting the lower rib cage and the gluteal folds [11]. The contemporary view of lower back pain is that it is a chronic condition as opposed to a self-limiting condition and should be treated and managed as a lifelong process.

Spinal manipulation therapy (SMT) is a form of therapy where practitioners use their hands or other devices to apply a controlled thrust to a particular joint of the spine [12]. SMT is typically performed by chiropractors. It has been proven in helping with chronic pain as an alternative form of medicine. In a randomized controlled trial conducted among 110 patients, patients were assigned either to a spinal manipulation group or a minimal manipulation group to assess pain sensitivity. In the study, patients received SMT and placebo SMT six times over two weeks [12]. The study assessed pain sensitivity prior to and immediately after the first intervention. The study revealed that SMT yielded a greater heat response from quantitative sensory testing, which demonstrated a reduction in pain sensitivity [12]. This study indicated that attenuation of pain sensitivity is greater in response to SMT than placebo SMT [12].

Furthermore, a controlled pragmatic trial aimed to assess the impact of SMT with at-home exercise and advice (HEA) and at-home exercise and advice alone in leg pain in the short and long term in adults with back-related leg pain [13]. Adults aged 21 years and older were enrolled. Patients were given 12 weeks of SMT along with HEA or only HEA. Patients were assessed at 12 weeks and 52 weeks [13]. In total, 191 patients of the 192 enrolled patients provided follow-up data at 12 weeks and 179 patients at 52 weeks [13]. The results of the study indicated that for leg pain, SMT with HEA had a clinical advantage at 12 weeks, but less of an impact at 52 weeks [13]. The study revealed that when patients received SMT with HEA after 12 weeks, there was an improvement in back-related leg pain. The study reported a significant benefit in treating chronic pain with SMT [13].

Furthermore, a study by Krekoukias et al. assessed the effectiveness of spinal mobilization compared to conventional physiotherapy [14]. There were three groups in this study, with the first group receiving five treatments of spinal mobilization, the second group receiving five sham treatments, and the third group receiving five treatments of conventional physiotherapy [14]. Each group consisted of 25 participants [14]. The conventional physiotherapy group included stretching exercises, transcutaneous electrical nerve stimulation, and massage. The assessment tool used in this study was rating pain intensity using a numerical pain rating scale and self-reported disability using the Oswestry and Roland-Morris Questionnaire at baseline and after the completion of the five treatment sessions [14]. When the data were collected and assessed it was seen that both the spinal manipulation group and the conventional physiotherapy group showed improvement in the pain rating scale [14]. When comparing the two groups, those who received the SMT showed a 75% improvement, whereas those in the group that received conventional physiotherapy showed a 25% improvement [14]. This data indicated that the use of spinal manipulation is effective in reducing lower back pain. It also indicated that it is a more effective tool in treatment compared to conventional physiotherapy [14].

A randomized, double-blind controlled trial was conducted to determine the efficacy of six osteopathic manipulative treatment (OMT) sessions over eight weeks [15]. The recovery was assessed in the 12th week using a composite measure of pain recovery and functional recovery. Relative risk and number needed to treat for recovery with OMT were measured and compared according to the patient’s baseline lower back pain intensity and functions [15]. In total, 345 patients did not meet the recovery criteria at baseline during the primary analysis, and 433 patients met at least one of the criteria in the sensitivity analysis [15]. A significant finding of the interaction of OMT and comorbid depression was noted. This gave an indication that patients without depression were more likely to recover from chronic lower back pain with OMT than those with depression [15]. OMT showed a significant measure of increased recovery from lower back pain [15].

A study was conducted to compare the change in temporal summation of heat pain (TSP) between SMT and spinal mobilization (MOB) in 92 healthy volunteers [16]. Patients were randomized to receive SMT, MOB, or no treatment for one session. The treatment involving SMT and MOB showed better results for decreased pain compared to no treatment [16]. During the study, the TSP procedure started in either the upper or lower extremities, which were randomly assigned. In the treatments that involved the legs, time was dependent on the treatment, and SMT and MOB showed an increased effect when compared to the change over time of the control group [16]. SMT and MOB differed from the control group but did not differ from each other. No treatment effect was observed in the upper extremities, such as the hands, suggesting that the effect was localized. The study provided support for a model, where joint-based manual therapies engage similar central nervous system mechanisms that lead to therapeutic benefits [16].

A randomized controlled trial conducted in 2013 aimed to determine the effective dose of spinal manipulation for chronic lower back pain [17]. The trial included 100 participants with chronic lower back pain who were randomized into four dose levels of care, namely, 0, 6, 12, or 18 sessions of spinal manipulation delivered by a chiropractor over six weeks [17]. Primary outcomes were measured on a 100-point modified Von Korff Pain Intensity and Functional Disability Scale and compared at 6, 12, 18, 24, and 52 weeks [17]. Overall, the pain was significantly reduced over the 52-week period, including for those who did not receive any SMT. The control group did not receive any treatment and showed an average of 15.7-point improvement in pain at the end of 52 weeks [17]. The treatment groups receiving 6, 12 and 18 treatments showed on average 19.3, 19.7, and 22.8 point improvement in pain intensity, respectively, after 52 weeks [17]. Furthermore, there was a significant decrease in functional disability as well with the control, 6, 12, and 18-session treatment groups showing a decrease of 17.2, 22.2, 23.7, and 26.1 in the functional disability scale, respectively, after 52 weeks [17]. The Functional Disability scale assessed the patients’ pain outcome on a scale of 0 (no pain) to 10 (extreme pain). The questionnaire asked about pain in various aspects, and, in the end, a total score was given. A score above 20 was considered moderate pain [17]. Secondary outcomes, such as pain unpleasantness, days with pain, days with a disability, perceived pain change, perceived disability change, physical health component, mental health component, health state, and non-prescription medication use were also measured during the course of treatment [17]. Overall, the study concluded that 12 sessions of spinal manipulation over six weeks yielded the best results for pain and functional disability improvement for chronic lower back pain. Furthermore, the study demonstrated that the results were sustainable after 52 weeks [17].

In 2017, Goertz et al. conducted a randomized controlled clinical trial to study the efficacy of chiropractic manipulation in the treatment of lower back pain [18]. In the study, patients were assigned to one of three cohorts: only receiving medical care, receiving medical and chiropractic care, or receiving medical and chiropractic care with additional collaboration. Patients were required to have experienced at least one month of lower back pain and were required to rate their pain on an 11-point scale [18]. A total of 131 subjects participated in this trial. The study showed improvements in pain and back-related disability in each of the three groups. Of these groups, none appeared to be statistically significant from the other groups in the measurements [18]. However, the two groups that received chiropractic manipulation experienced a much higher improvement in perceived pain and were more satisfied with their treatment compared to the group that received only medical management [18].

Acupuncture

Acupuncture is a form of medicine that has been used for several years and originated in China over 3,000 years ago. The use of acupuncture has been beneficial for many years in the treatment of chronic pain [19]. A type of acupuncture therapy, thread-embedding acupuncture affects the tissue both mechanically and chemically and involves a thread that can continuously stimulate the acupuncture points [20].

A randomized, controlled, blinded trial involving 38 patients with chronic lower back pain showed that the treatment was safe and effective via a significant reduction in the visual analog scale. In this assessor-blinded study, 38 patients with chronic lower back pain for over three months were randomly divided into two parallel groups [20]. All participants received acupuncture therapy treatment twice a week for eight weeks. The treatment group received one additional thread-embedding acupuncture treatment per week. The participants were not taking analgesics for the duration of their treatment to avoid confounding variables. The patients answered surveys every two weeks and had a final assessment conducted by the blinded assessors three months after the final treatment [20]. The assessors measured the efficacy of the treatment primarily on the visual analog scale and with minimal clinically important difference [20]. Results showed that both the treatment and control groups showed significant improvement in the participant’s chronic lower back pain; however, the treatment group receiving acupuncture therapy and thread-embedded acupuncture therapy demonstrated faster analgesic effects [20]. The results suggested that though the treatment group may have shown 50% improvement by rapid therapeutic effects that were maintained until the 20-week follow-up, and the control group only showed 30% improvement, its proportion increased to similar levels of the treatment group by the 20-week follow-up, yielding similar results [20]. The study demonstrated that acupuncture therapy and acupuncture therapy with thread-embedded acupuncture is almost equally as effective in the treatment of chronic lower back pain, with both showing continuous effects even after treatment had concluded [20].

A systematic review of 29 randomized controlled trials was conducted to evaluate acupuncture as a treatment for chronic pain [10]. The systematic review concluded that acupuncture is the best holistic approach for treating patients with neck and lower back pain, osteoarthritis, headaches, and shoulder pain [10]. Acupuncture improved pain scores, measures of disability, and depression. These results remained consistent even after excluding randomized controlled trials with a risk of potential bias [10]. It was also reported that acupuncture can be combined with other holistic therapies for the treatment of lower back pain patients to help improve function. Acupuncture was also shown to be well-tolerated and safe with no significant adverse reactions within the conducted trials [10].

Another study compared the effect of laser acupuncture (LA) and electroacupuncture (EA) in patients with knee osteoarthritis. EA and LA have been known to reduce pain in knee osteoarthritis patients [21]. A total of 50 patients diagnosed with knee osteoarthritis, 50 years or older, and with consistent knee pain for over three months were randomly assigned to the treatment group, EA plus LA, or the control group, EA plus sham LA without laser output [21]. All patients in the treatment group underwent a combined EA and LA treatment three times a week for four weeks. The subjects in the control group underwent the same treatment as the treatment group, except the patients were not exposed to any laser output. The outcome measurements included a visual analog scale, Western Ontario McMaster Universities Osteoarthritis Index, knee injury and osteoarthritis outcome, body composition analysis, knee range of motion, quadriceps muscle stiffness, one leg standing with eyes open test, and a chair stand test before and after four weeks of intervention [21]. LA is painless, non-invasive, has no risk of infection, and can be combined with normal, traditional acupuncture [21]. LA is mostly used to address arthralgia or muscular pain. Clinical effects of LA encompass anti-inflammatory, neural modulation, and cell healing and regeneration effects. Both LA and EA have anti-inflammatory and neural modulation effects that lead to decreased knee pain in knee osteoarthritis [21].

Yoga

A randomized trial was conducted to compare the effectiveness of yoga and physical therapy on chronic lower back pain. Both yoga and physical therapy were effective in patients with functional disability and pain, but this study aimed to determine if yoga is non-inferior to physical therapy [22]. A total of 320 participants with non-specific chronic lower back pain were divided into three randomized groups where one group received yoga intervention, the second group received physical therapy intervention, and the third group received educational literature [22]. The pain was measured on an 11-point scale at 12 weeks after completion of the intervention. The results demonstrated the non-inferiority of yoga to physical therapy for the treatment of non-specific chronic lower back pain. Furthermore, the improvements seen in both yoga and physical therapy intervention groups were maintained for one year, and the frequency of adverse events did not differ between both groups [22].

In a systematic review evaluating the effects of yoga on patients with chronic non-specific neck pain, 10 randomized control trials comparing the effects of yoga with other interventions were included in the analysis [23]. The review primarily focused on pain and disability and evaluated other outcomes such as range of motion, quality of life, and mood. Moreover, the studies being reviewed had different sessions and durations of yoga intervention, and the analysis was unable to compare the effects of different types of yoga styles. All included studies in the analysis evaluated immediate and short-term effects of yoga treatments, but only two of the included studies evaluated beyond six months, leading to the conclusion that yoga is effective in the treatment of chronic non-specific neck pain to provide more short-term relief compared to long-term treatment [23]. Overall, the results of the analysis showed that yoga had a positive effect on neck pain intensity along with other secondary outcomes. The authors concluded that yoga could significantly relieve neck pain intensity and could be a positive alternative treatment for chronic non-specific neck pain [23]. The results obtained throughout the analysis are presented in Table 1.

**Table 1:** Summary of the studies showing the outcomes of the analyzed integrative medicine techniques.

Type of integrative medicine	Year	Journal	Authors	Type of study	Sample size	Conclusion
Massage	2015	The Spine Journal	Cook et al. [8]	Randomized clinical trial	228	Participants in the 60-minute massage intervention group showed a significant reduction in chronic neck pain
Massage	2017	Primary Care: Clinics in Office Practice	Hillinger et al. [10]	Review	25	Patients mostly benefited from massage therapy intervention for chronic low back pain. There were no adverse effects associated with massage
Massage	2018	International Journal of Therapeutic Massage and Bodywork: Research, Education, & Practice	Massingill et al. [9]	Pilot randomized controlled trial	21	Myofascial massage is effective in the management of pain in patients post-breast cancer surgery. It also has benefits in mobility limitations
Osteopathic spinal manipulation	2014	Journal of Pain	Bialosky et al. [12]	Randomized controlled trial	110	Patients underwent a pain sensitivity study, where spinal manipulation, in comparison with a placebo, yielded a greater heat response, which showed a reduction in pain sensitivity
Osteopathic spinal manipulation	2014	Annals of Internal Medicine	Bronfort et al. [13]	Trial with adaptive allocation	192	For patients with leg pain, spinal manipulation therapy with at-home exercise and clinical advice at 12 weeks had less of an impact than that at 52 weeks. There was also an improvement in back-related leg pain
Osteopathic spinal manipulation	2014	The Spine Journal	Haas et al. [17]	Randomized controlled trial	100	Chronic lower back pain was significantly improved with 12 sessions of spinal manipulation over six weeks. Functional disability also showed signs of improvement
Osteopathic spinal manipulation	2016	Journal of Osteopathic Medicine	Licciardone et al. [15]	Randomized controlled trial	433	The study reported a significant finding of interaction between osteopathic spinal manipulation and depression. Patients without depression were more likely to recover from chronic lower back pain with osteopathic spinal manipulation than those with depression
Osteopathic spinal manipulation	2017	Journal of Manipulative Therapy	Krekoukias et al. [14]	Randomized controlled trial	75	Patients in the spinal manipulation intervention group showed a 75% improvement in chronic lower back pain compared to the physical therapy treatment group which only showed a 25% improvement
Osteopathic spinal manipulation	2017	The Journal of Pain	Penza et al. [16]	Randomized controlled trial	92	Spinal manipulation therapy showed equally as effective results as spinal mobilization in the localized reduction of temporal summation of heat pain
Osteopathic spinal manipulation	2017	BMC Geriatrics	Goertz et al. [18]	Randomized controlled trial	131	Patients in the study received three forms of treatment. Of the three, the two groups receiving the treatment with chronic manipulation showed improvement when compared to those that received medical management alone
Acupuncture	2017	Primary Care: Clinics in Office Practice	Hillinger et al. [10]	Systematic review	25	Acupuncture the best holistic approach for the treatment of chronic pain and improves disability and depression as well. It is well tolerated, safe, and has few adverse effects
Acupuncture	2020	Medicine	Sung et al. [20]	Randomized controlled trial	38	Acupuncture therapy and acupuncture therapy with thread-embedded acupuncture are almost equal in effectiveness in the treatment of chronic low back pain. Both exhibit continued effectiveness even after the conclusion of therapy
Acupuncture	2020	Medicine	Wu et al. [21]	Randomized controlled trial	50	Laser acupuncture and electroacupuncture were found to have anti-inflammatory and neural modulation effects that led to decreased knee pain in patients with knee osteoarthritis
Yoga	2017	Annals of Internal Medicine	Saper et al. [22]	Randomized controlled trial	320	Yoga demonstrated non-inferiority to physical therapy in the reduction of chronic low back pain. In addition, improvements were maintained after one year
Yoga	2019	Medicine	Li et al. [23]	Systematic review, meta-analysis	10	Yoga is effective in the treatment of chronic non-specific neck pain, reducing the intensity of pain, along with other secondary outcomes such as range of motion and quality of life

## Conclusions

Chronic pain affects millions of patients daily. Current treatments usually include opioid therapy and NSAIDs. However, chronic opioid use carries a fairly immense risk, given the potential for overdose and addiction with this specific class of medications. The outcomes of this review can be used to aid in the treatment of chronic pain using integrative medicine. Out of 27 articles, 15 were selected based on the inclusion and exclusion criteria for the review on chronic pain. In future studies, a more concise review consisting of randomized controlled trials that evaluate the short- and long-term effects would yield a better review.

Massage therapy has been analyzed in this study to understand its effects and benefits in dealing with chronic pain. In all trials analyzed in this study, massage therapy showed decreased pain compared to pretreatment. The areas that were treated with massage therapy in this study were the lower back, neck, and breast. The results of the trials of massage therapy and chronic pain showed significant evidence that massage therapy can be an adequate replacement for the pharmaceutical management of chronic pain.

The use of acupuncture has been around for many years, but only recently has it become an area of interest in treating chronic pain. From the research that has been conducted, acupuncture is an appropriate approach to treating chronic pain with beneficial results. EA and LA were also proven to be significantly effective in treating chronic pain involving patients with knee osteoarthritis.

Through systematic evidence of yoga being used for chronic pain, yoga was significantly effective when treating chronic neck pain. The combination of physical therapy and yoga also showed a significant reduction in pain.

Despite the small sample size of the articles included in this review, we can conclude that this review showed a significant reduction in chronic pain when using alternative approaches such as massage therapy, osteopathic and spinal manipulation, acupuncture, and yoga. All forms of alternative medicine allowed patients to achieve pain relief without using opioids and NSAIDs.
